# Comparison of condylar inclination angle in edentulous subjects: CBCT versus protrusive interocclusal record

**DOI:** 10.6026/973206300212664

**Published:** 2025-08-31

**Authors:** Priyanka Chopra, Kavipal Singh, Nimish Sethi, Balwinder Singh, Neelam Suman, Ramninder Bawa

**Affiliations:** 1 Department of Prosthodontics and Crown and Bridge, Sri Guru Ram Das Institute of Dental Sciences and Research, Sri Amritsar, Punjab, India; 2 Department of Oral Diagnosis, Oral Medicine & Radiology, Sri Guru Ram Das Institute Of Dental Sciences and Research, Sri Amritsar, Punjab, India

**Keywords:** CBCT, condylar guidance, cross-sectional view, panoramic view, protrusive interocclusal record

## Abstract

Horizontal condylar guidance angles obtained using two different methods: the clinical protrusive interocclusal record method and the radiographic method using CBCT (Cone Beam Computed Tomography). The key issue is the observed significant differences in the condylar guidance angles measured by the clinical method and those measured radiographically, with the radiographic measurements being consistently higher than the clinical ones. A total of 25 completely edentulous individuals were included, with condylar guidance angles measured clinically using the protrusive interocclusal record method and radiographically through CBCT in two formats. Results showed significant differences in the angles between the methods, with radiographic measurements being higher than clinical values. Strong correlations were observed between the clinical process and the CBCT panoramic and cross-sectional views. The study concludes that CBCT measurements provide more precise condylar guidance angles than the protrusive interocclusal record method.

## Background:

For effective prosthodontic rehabilitation, harmony between the patient's stomatognathic system and the prosthesis is crucial [1]. Condylar inclination, a key factor in Hanau's quint, governs balanced articulation and accurate simulation of the condylar path on an articulator is essential [2]. Condylar guidance, defined as the mandibular guidance generated by the condyle and articular disc along the glenoid fossa, ensures proper occlusion [3, 4]. Misalignment of condylar guidance can lead to occlusal interferences, causing issues like periodontal trauma, muscle spasms and TMJ pain, as well as increasing chairside adjustment time, leading to patient dissatisfaction [5, 6]. Previous studies have compared cephalometric and gnathologic methods for measuring the condylar head's relation to the articular eminence, with strong correlations between protrusive interocclusal records and radiographic images [5]. CBCT has improved the accuracy, safety and convenience of radiographic measurements [7, 8]. Therefore, it is of interest to correlate clinical and CBCT-based horizontal condylar guidance angles to enhance measurement accuracy and reduce occlusal complications, ultimately improving prosthodontic outcomes.

## Materials and Methods:

The study was conducted on 25 patients to compare the horizontal condylar guidance angle obtained by extra oral gothic arc tracing using protrusive interocclusal records and by cone beam computed tomography. The study was conducted after receiving ethics committee approval and informed consent from the patient. Patients with good neuromuscular control, good mental health and undergoing CBCT for implant placement but who were later unwilling to undergo the implant procedure were included in the study. Patients with TMJ disorders, orofacial tumors, gross facial deformity, radiation therapy, parafunctional habits, systemic conditions and autoimmune disorders were excluded from the study. For the clinical horizontal condylar guidance registration, Primary impressions of the patients were made with impression compound (PYRAX; Pyrax Polymars, India). Primary casts were made and special trays were fabricated with autopolymerizing acrylic resin (DPI RR Cold cure, India). Border molding was then completed with the aid of green stick compound (DPI Pinnacle Tracking Sticks, India) and definitive impressions were taken using Zinc oxide Eugenol impression paste (DPI Impression Paste, India). Definitive impressions were then poured with Dental stone (Dentstone; Neelkanth Ortho Dent Pvt. Ltd., India) and definitive casts were made. Record bases were made with autopolymerizing acrylic resin (DPI RR Cold cure, India) over which occlusal rims was fabricated with modelling wax (PYRAX; Pyrax Polymars, India). Orientation jaw relation was recorded with Hanau Spring Bow (Figures 1, 2 - see PDF) and the maxillary cast was mounted on the Hanau Wide Vue semi-adjustable articulator. After taking a tentative vertical jaw relation, the mandibular cast was mounted. Another pair of record bases was made and impression compound rims were fabricated to which extraoral gothic arc tracers were attached. After an extraoral gothic arc tracing was made, a plaster interocclusal record was made at 6mm protrusion for each patient (Figures 3 - see PDF). The record was used to register the right and left condylar guidance values on the Hanau Wide Vue articulator.

## For the radiographic determination of horizontal condylar guidance:

A CBCT scan of each individual was obtained of volume 17.5cm X 13 cm. On each side, the Frankfort horizontal plane was drawn by joining the two landmarks - "orbitale" and "porion" and this was considered as the first constructed line. A second line representing the mean condylar path was constructed by joining the most superior point on the glenoid fossa and most inferior point of articular eminence. Sagittal condylar guidance angle was obtained by measuring the angle formed between the intersection of the two constructed lines for both the right and the left sides. The angles were recorded in two formats - panoramic and cross section. To standardize the study, arch formation was done at 4.5 mm below the plane of the superior part of the condyle. The CBCT panoramic section was determined with a sectioning line at the middle part of the both condyle and the nasal tip and the CBCT cross section were determined by sectioning at the center of the condyle cross section. The data recordings comprised of three sets of values for all participants; the right and left horizontal condylar guidance angle obtained by protrusive interocclusal records and right and left horizontal condylar guidance measured with CBCT panoramic and CBCT cross sections. Data thus obtained was analyzed statistically.

## Results:

The condylar angles recorded by clinical and radiographic methods were compared. The data thus obtained was put to statistical analysis and the results were obtained using t-test, Pearson's correlation test, one-way ANOVA test and Tukey HSD post Hoc test. The results obtained in statistical t-test comparisons of the methods between the right and left sides are presented in Table 1 (see PDF). One way ANOVA test showed highly significant differences between clinical, CBCT Panoramic and CBCT cross section methods for both left and right sides as seen in (Tables 2, 3 - see PDF). Post hoc paired comparison (Table 4 - see PDF) showed that when comparing the three methods, the condylar guidance angle was significantly higher for the CBCT panoramic and CBCT cross section method than for the protrusive occlusal record. Pearson's correlation test comparisons between the methods (Table 5 - see PDF) showed strong correlations between the measurements using CBCT Panoramic, CBCT cross section and the protrusive occlusal record method. The reliability of the measurements was confirmed using Cronbach's α value.

## Discussion:

Condylar guidance is described as the mandibular guidance generated by the condyle and articular disc traversing the contour of the glenoid fossa which can be simulated mechanically in the articulator [9]. Various methods can be used to determine the horizontal condylar inclination, such as interocclusal records, radiographic methods, pantographic tracings and electronic jaw tracking devices [8]. Establishing horizontal condylar guidance angle using protrusive interocclusal records is a multistep procedure which requires expertise and experience of the clinician. Thus, many dental practitioners use average values for setting horizontal condylar guidance angles to avoid the clinical method which may result in inaccuracies and interferences which can hamper the balanced occlusion if the horizontal condylar path inclination is very flat or very steep [9]. In the present study, mean horizontal condylar guidance values obtained from clinical method for left side was 36.80 degrees and right side was 36 degrees. The mean horizontal condylar guidance values obtained from CBCT panoramic method for left side was 44.36 degrees and right side was 41.96 degrees and the mean horizontal condylar guidance values obtained from CBCT cross section method for left side was 44.92 degrees and right side was 43.04 degrees. Thus, showing that radiographic method yielded greater value of condylar guidance angles as compared to clinical method; this is in accordance to the observations of Shrestha *et al.* (2012) [10] who also found higher horizontal condylar guidance values with CT scan recording than with the clinical methods.

The results obtained were also consistent with the study by Prasad *et al.* (2012) [11] who found that the condylar guidance values obtained by radiographic method was greater by 1.97 degrees and 3.18 degrees than the protrusive interocclusal record method for right and left sides. The study found a strong degree of correlation between the condylar guidance angles determined by both methods and concluded that keeping in mind the inaccuracies of the interocclusal record technique with inherent errors up to 30°; the radiographic method may have clinical relevance. It was further noted that with respect to mean change in value of angles, there existed highly significant differences between clinical, CBCT panoramic and CBCT cross section methods for both right and left sides. It was further inferred that strong correlations exist between the measurements using CBCT panoramic, CBCT cross section and the protrusive occlusal record method. Cronbach's α was high for all of the methods, indicating internal consistency. Therefore, measuring the condylar guidance angle using radiographic images might indeed be a useful method; a clinically applicable condylar guidance angle can be obtained by adjusting the value measured using radiographic images. The CBCT panoramic values were higher by 7.56 degrees for left and by 5.96 degrees for right side. The CBCT cross section values were higher by 8.12 degrees for left and by 7.04° degrees for right side. Thus, CBCT measurements of the condylar guidance angles were 6 - 8 degrees higher than the protrusive occlusal record measurements recorded. Singh *et al.* (2017) advocated for clinical method as radiographic method yielded a consistent greater value as compared to clinical method [2]. A panoramic radiographic image in the temporal region shows the outer radio-opaque line depicting the articular eminence and inner radiopaque line depicting the inferior border of the zygomatic arch. Panoramic radiographs can show errors due to patient positioning. In the present study cone beam computed tomography has been used which provides a three-dimensional image for both the sides without superimpositions and so the glenoid fossa and the articular eminence can be clearly delineated. On the other hand, with the advent of cone beam computed tomography, scans have become safer involving lesser radiation exposure and more accuracy resulting in their widespread application in dentistry. In a study by Mawani *et al.* (2019) it was concluded that the values for condylar guidance obtained from CBCT and OPG are comparable and correlated. CBCT being a better radiographic technique can be used for obtaining the condylar inclination for programming the semi adjustable articulators [4]. Therefore, it can be inferred from this study that the horizontal condylar guidance angle obtained by clinical or gothic arc tracing technique is not comparable with that obtained radiographically. However, there is a strong correlation between the clinical method, CBCT panoramic and CBCT cross-section method so the radiographic technique should not be omitted and the values obtained can be modified to program the articulator. Thus, this study infers that subtracting 8 degrees from the condylar guidance angles measured using CBCT images seems to be reasonable.

## Conclusion:

Significant differences were found between radiographic imaging methods and the protrusive occlusal record method for condylar guidance angles. CBCT panoramic and cross-sectional values showed no significant difference and radiographic values were consistently higher than those obtained clinically. Subtracting 6-8 degrees from CBCT measurements is recommended for clinical applications, simplifying prosthodontic treatment.
